# Supplementary material for: Receptor transporter protein 4 (RTP4)-mediated repression of hepatitis C virus replication in mouse cells
Source: PLoS Pathog. 2025 Sep 8;21(9):e1013412. doi: 10.1371/journal.ppat.1013412 (PMC12431671; doi:10.1371/journal.ppat.1013412)
Supplement: S1 Fig — A-B. AlphaFold structural predictions of A. hsRTP4 and B. mmRTP4. C. MatchMaker superimposition of structures in A and B. D-E. AlphaFold structural predictions of D. hsRTP4mmDVR and E. mmRTP4hsDVR. F. MatchMaker superimposition of structures in D and E. G-L. AlphaFold structural predictions of G. hsRTP4mmZFD_1, H. hsRTP4mmZFD_2, I. hsRTP4mmZFD_3, J. mmRTP4hsZFD_1, K. mmRTP4hsZFD_2, L. mmRTP4hsZFD_3. (DOCX) [file ppat.1013412.s001.docx]

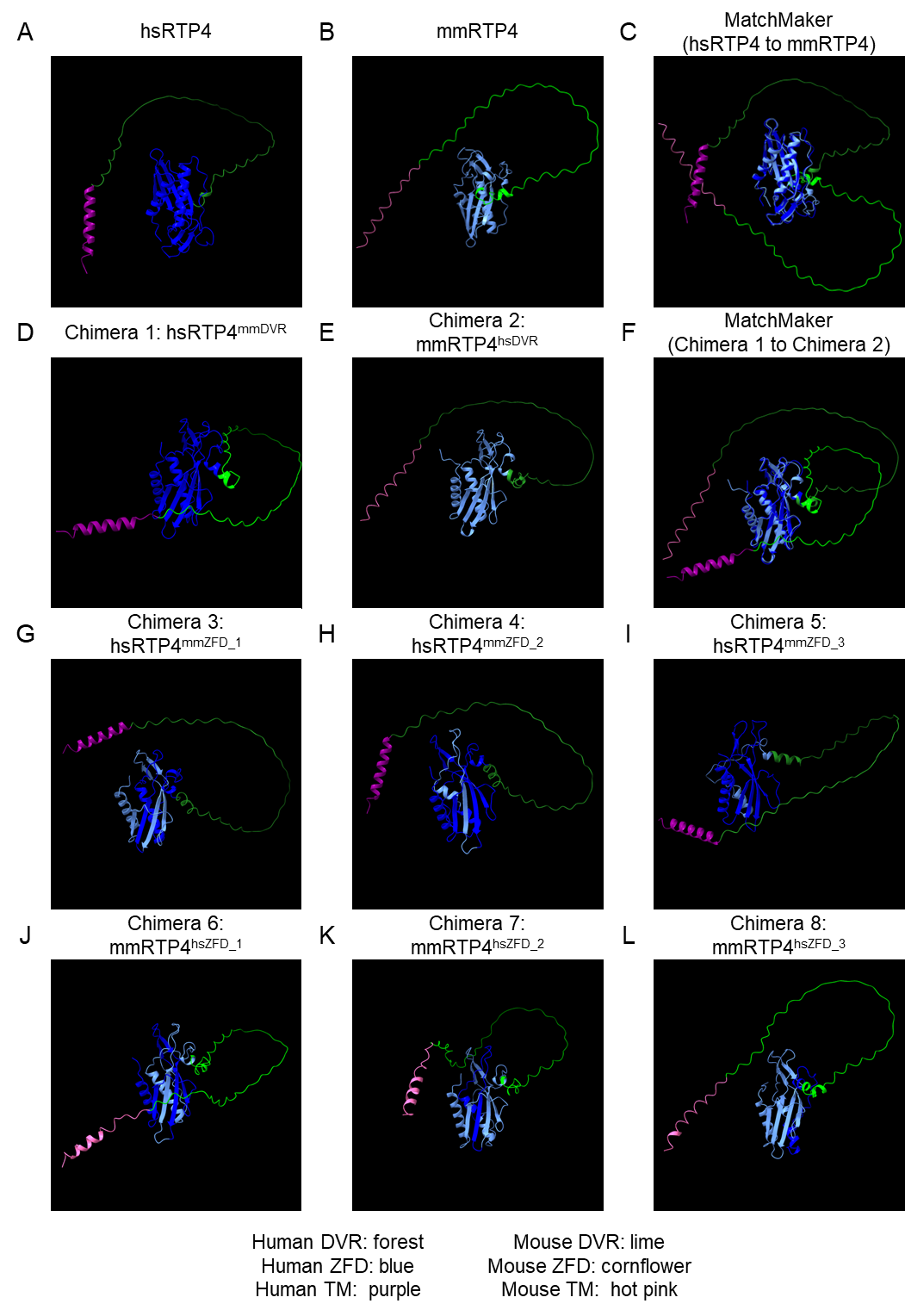


**Fig. S1 | Structural prediction of RTP4 chimeras. A-B.** AlphaFold structural predictions of **A.** hsRTP4 and **B.** mmRTP4. **C.** MatchMaker superimposition of structures in **A** and **B**. **D-E.** AlphaFold structural predictions of **D.** hsRTP4^mmDVR^ and **E.** mmRTP4^hsDVR^. **F.** MatchMaker superimposition of structures in **D** and **E**. **G-L.** AlphaFold structural predictions of **G.** hsRTP4^mmZFD_1^, **H.** hsRTP4^mmZFD_2^, **I.** hsRTP4^mmZFD_3^, **J.** mmRTP4^hsZFD_1^, **K.** mmRTP4^hsZFD_2^, **L.** mmRTP4^hsZFD_3^.
