# Supplementary material for: Receptor transporter protein 4 (RTP4)-mediated repression of hepatitis C virus replication in mouse cells
Source: PLoS Pathog. 2025 Sep 8;21(9):e1013412. doi: 10.1371/journal.ppat.1013412 (PMC12431671; doi:10.1371/journal.ppat.1013412)
Supplement: S2 Fig — A. Transfection of FLAG-tagged RTP4 bicistronic expression constructs in 293T cells. B. Quantification of bicistronic fluorophore expression in transfected samples. C. RT-qPCR analysis of samples using primers targeting FLAG and gene-specific sequences. Blue, FLAG-hsRTP4 RNA; green, FLAG-mmRTP4 RNA; ND, not detected. D. Western blot analysis of cell lysates using mouse anti-FLAG and rabbit anti-β-actin; this gel is utilized in Fig 5B. Some figure elements (mouse [75], liver [76]) were sourced from the public domain and are listed as references. (DOCX) [file ppat.1013412.s002.docx]

**Fig. S2 | Quantification of FLAG-RTP4 expression via flow cytometry, RT-qPCR, and Western blot. A.** Transfection of FLAG-tagged RTP4 bicistronic expression constructs in 293T cells. **B.** Quantification of bicistronic fluorophore expression in transfected samples. **C.** RT-qPCR analysis of samples using primers targeting FLAG and gene-specific sequences. Blue, FLAG-hsRTP4 RNA; green, FLAG-mmRTP4 RNA; ND, not detected. **D.** Western blot analysis of cell lysates using mouse anti-FLAG and rabbit anti-β-actin; this gel is utilized in Figure **5B**. Some figure elements (mouse [[75]](https://paperpile.com/c/S3D3EQ/SeMH), liver [[76]](https://paperpile.com/c/S3D3EQ/Xb1N)) were sourced from the public domain and are listed as references.

**
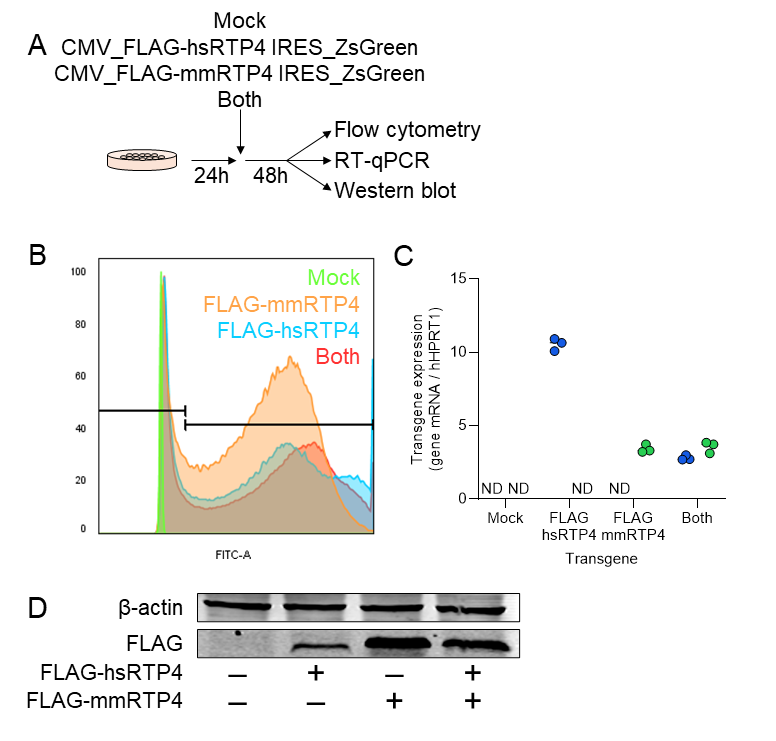
**
