# Supplementary material for: Receptor transporter protein 4 (RTP4)-mediated repression of hepatitis C virus replication in mouse cells
Source: PLoS Pathog. 2025 Sep 8;21(9):e1013412. doi: 10.1371/journal.ppat.1013412 (PMC12431671; doi:10.1371/journal.ppat.1013412)
Supplement: S3 Fig — A. Schematic of FLAG-hsRTP4 transfection in Huh7 Lunet cells. B. RT-qPCR quantification of FLAG-hsRTP4 transgene expression in samples prepared in A. ND, not detected. C. Schematic of mmRTP4 induction in primary murine hepatocytes (PMH) in vivo. D. RT-qPCR quantification of FLAG-hsRTP4 transgene expression in samples prepared in D. (DOCX) [file ppat.1013412.s003.docx]

**
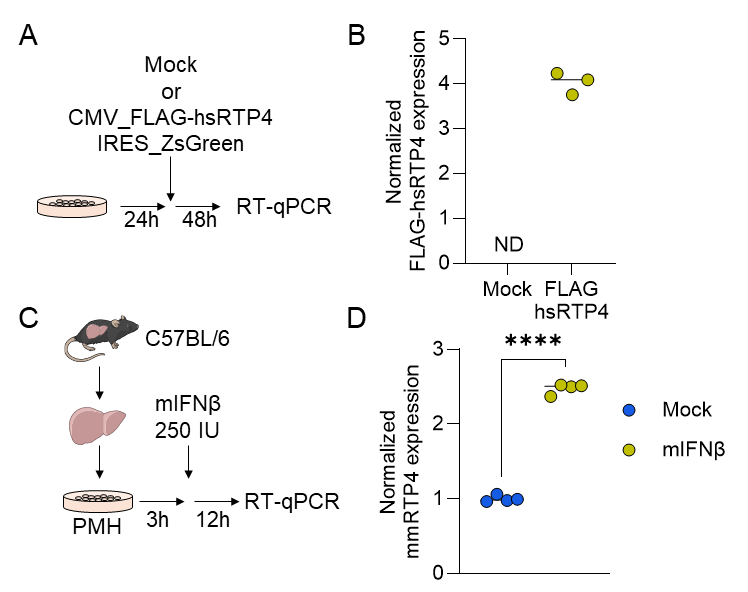
**

**Fig. S3 | hsRTP4 ectopic expression *in vitro* is comparable to induced mmRTP4 expression in *ex vivo* tissue. A.** Schematic of FLAG-hsRTP4 transfection in Huh7 Lunet cells. **B.** RT-qPCR quantification of FLAG-hsRTP4 transgene expression in samples prepared in **A**. ND, not detected. **C.** Schematic of mmRTP4 induction in primary murine hepatocytes (PMH) *in vivo*. **D.** RT-qPCR quantification of FLAG-hsRTP4 transgene expression in samples prepared in **D**.
