# Supplementary material for: Receptor transporter protein 4 (RTP4)-mediated repression of hepatitis C virus replication in mouse cells
Source: PLoS Pathog. 2025 Sep 8;21(9):e1013412. doi: 10.1371/journal.ppat.1013412 (PMC12431671; doi:10.1371/journal.ppat.1013412)
Supplement: Attachment — Submitted filename: RTP4 Manuscript Rebuttal.docx [file ppat.1013412.s005.docx]

First, we would like to thank all reviewers for taking the time to provide such thorough and extensive reviews. We recognize and appreciate the effort that went into these, and we seek to provide explanations and experimental data to address the many valid points raised in their reviews.

**Point-by-point rebuttal**

**Major Issues**

***Reviewer #1:***

*1. Figure 2: The observation that mmRTP4hsDVR exhibited reduced antiviral activity compared to mmRTP4 suggests that the DVR-TM region is necessary but not sufficient for antiviral activity, as hsRTP4mmDVR lacked antiviral effects. Conversely, mmRTP4hsZFD_1 retained full activity, indicating that the C-terminal ZFD is both necessary and sufficient for antiviral function. Thus, based on these data, to this reviewer, the C-terminal part of ZFD and the DVR-TM are important for antiviral activity. Utilizing AlphaFold-predicted structures could provide further insights into these functional domains.*

**Response:** We thank the reviewer for this insightful comment. We have included figures of AlphaFold structures in the supplementary data of our resubmission, including color-coded models for human RTP4, murine RTP4, and all 8 chimeras generated. We further include MatchMaker analyses to quantify structural similarities between murine and human RTP4, as well as between chimeras 1 and 2. We note that the DVR is not well-resolved by AlphaFold modeling (given its disordered nature), precluding structural insights into it specifically. We have updated the text of the manuscript to reference these data.

*2. Figures 3C and 3D: The experimental details require clarification. Specifically, it is unclear whether cells were passaged during the 9-day period following HCVcc infection and lentiviral transduction. Given the sensitivity of HCV replication to cellular confluency, the observed phenotypes may be influenced by cell state. Additionally, the reduction in intracellular virus titer (Figure 3E) was less pronounced than the decrease in NS5A+ cells (Figure 3C), suggesting that mmRTP4 may primarily affect NS5A protein levels through translational or post-translational mechanisms. The reviewer disagrees with the conclusion that mmRTP4 inhibits infectious particle formation, as viral titers in the supernatant were comparable across groups.*

**Response:** We thank the reviewer for this comment; we have clarified the timeline for this experiment in the text, indicating that cells were not passaged during the indicated 9-day period We note that the data in 3C and 3D are likely not reflective of cell state, given the lack of decrease in NS5A^+^ cells in the mock-transduced (purple) cohort. We have separately toned down language regarding the conclusions from figure 3E to reflect the second half of the reviewer’s comment.

*3. Figure 5: The rationale for selecting a highly sensitive method to detect RTP4-NS5A interactions should be explained. The use of complementary techniques, such as co-immunoprecipitation (co-IP) or co-localization studies, would strengthen the findings. Additionally, demonstrating RTP4’s interaction with HCV replication complexes (RCs) through dsRNA detection using anti-dsRNA antibodies could provide further mechanistic insights. Or the MOA is the same as reported, that RTP4 binds to viral genome?*

**Response:** We thank the reviewer for this comment, and we have added text to explain our choice of assay for protein-protein interactions (PPIs). We agree that the proximity ligation assay (PLA) provides excellent, robust evidence of protein-protein interactions due to the closeness of interactors required for rolling-circle amplification (Bagchi, Fredriksson, & Wallén-Mackenzie (2015) Methods in Molecular Biology, vol 1318). We opted for this highly sensitive method due to limited empirical utility of the anti-JFH1 NS5A antibody for pull-down assays. Regarding an immunofluorescence-based colocalization study, the PLA relies on the same type of colocalization, only with a higher bar of sensitivity (given spinning-disc pixel size versus the radius required for PLA). We have attempted extensively to detect HCV dsRNA in infected cell-culture, but these assays have proven prohibitively difficult in even highly infected cell-culture samples. Based on the established mechanism for black flying-fox RTP4 against flaviviruses and human RTP4 against coronaviruses, we propose a putative mechanism wherein RTP4 binds to viral genome, sterically hindering replication; this is included in our Discussion section.

*4. Figure 6B: It is unclear whether viral replication was detectable in the CD81EL2[H/H] OCLNEL2[H/H] control group. This information is critical for interpreting the results.*

**Response:** We thank the reviewer for this comment – in general, HCV uptake, but not replication, is detected in EFKI mice, as our group has shown previously (Ding et al. 2017 J Virol). We have clarified this in the text of our resubmission. We note that attempts to detect HCV replication *in situ* are notoriously difficult (Pal et al. (2006) J Virol; Kandathil et al. (2013) Gastroenterology; Liang et al. (2009) Gastroenterology; Stiffler et al. (2009) PLoS One). While we have attempted to detect minus-strand genome in *ex vivo* tissue samples, these attempts have been unsuccessful even in mice demonstrating high levels of HC viremia.

*5. Figure 7: The absence of a positive control, such as IFN receptor knockout (IFNR KO) mice, limits the interpretability of the data. Including such controls would enhance the robustness of the findings.*

Response: We thank the reviewer for this suggestion, and agree that a positive control would strengthen the *in vivo* arm of this study. We do not have EFKI on an IFNAR background and hCD81 hOCLN STAT1-/- are only minimally permissive to HCV (e.g. Dorner et al. (2013) Nature). To demonstrate that the viral inoculum used is infectious *in vivo*, we have modified figure 7 to include data from a human liver-chimeric mouse infected with HCV-Jc1.

*6. Restriction Factor Phenotypes: The expected outcome of knocking out a restriction factor is enhanced viral replication. The reviewer suggests exploring the effects of hRTP4 knockout on human viruses and mmRTP4 knockout on murine viruses (e.g., murine hepatitis virus, MHV) to better understand the role of RTP4 in viral restriction.*

**Response:** We thank the reviewer for this excellent suggestion. As we indicate in the discussion section of this manuscript, separate publications have already been undertaken to explore the role of RTP4 in viral restriction in black flying foxes (against ZIKV and DENV) and humans (IAV, SARS-CoV-2, HCoV-OC43). We agree that the impact of RTP4 knockout upon MHV infection would be fascinating to explore, we do not have systems in our lab to produce this virus, nor do we believe that it is within the scope of this HCV-centered study. The differential permissiveness of our RTP4^-/-^ mouse line to other human pathogens is a topic of ongoing investigation.

***Reviewer #2:***

*1. RTP4 is an ISG, but the entire manuscript uses ectopic expression of hs- or mmRTP4 in human cells as a model. Still, it would be important to know, how the levels of ectopic expression, particularly of mmRTP4, compare to native expression levels in murine hepatocytes, in presence and absence of interferon induction. If no antibodies are available, RNA-data could be used as an alternative.*

**Response:** We thank the reviewer for this comment. To address this, we performed an experiment in which primary murine hepatocytes (PMHs) from C57BL/6 mice were stimulated for 12 hours with 250 IU murine IFNβ, followed by RT-qPCR analysis of RTP4 expression. This resulted in a ~2.5-fold increase in mmRTP4 expression. These are similar levels of expression to those observed in Huh7 Lunet cells transfected with FLAG-tagged human RTP4. These data are included in the supplementary data file of this resubmission.

We contend that ectopic overexpression of ISGs is an important system to analyze their function, as has been leveraged previously (Schoggins et al. 2014 Nature; Schoggins et al. 2011 Nature).

*2. The authors should provide bulk Western blot data to ensure similar expression of the RTF4 orthologues and chimeras, if antibodies are available, since the stability of the proteins might differ, particularly regarding the chimeras. This could at least be shown on protein level for the tagged variants used in Fig. 5.*

**Response:** We thank the reviewer for this suggestion. Unfortunately, all commercial antibodies against human or murine RTP4 fail to detect the protein in our hands, even in contexts of over-expression. In the supplementary file of this resubmission, we provide flow cytometry, RT-qPCR, and Western blot data from 293T cells transfected with the tagged RTP4 constructs in Figure 5, indicating stable expression. We include Western blot data in our resubmission as Figure 5B.

*3. Fig. 5 and associated text: The data indicate that RTP4 is in proximity to NS5A but neither that NS5A physically interacts nor that NS5A is the sole interacting protein in the HCV replicase, as claimed by the authors throughout the manuscript, including abstract. To support these claims, the authors need to include co-immunoprecipitation data (direct interaction) and use another protein of the replicase (e.g. NS3, NS4B or NS5B) as a control. Alternatively, the authors need to tone down their claims, e.g. by stating that NS5A was used as a marker for the replicase.*

**Response:** We thank the reviewer for this comment. Given a dearth of reliable antibodies for other HCV JFH1 nonstructural proteins, we agree to tone down our claims to instead state that “murine RTP4 comes into apposition with HCV NS5A” and “murine RTP4 associates with the replication organelle” rather than “murine RTP4 directly binds NS5A”. While we note that the radius for interaction via PLA is < 40 nm (Bagchi, Fredriksson, & Wallén-Mackenzie (2015) Methods in Molecular Biology, vol 1318), and the HCV replication organelle is approximately 186 nm in diameter (Stoeck et al. (2017) J Virol), we agree that a more measured assessment of our data may be beneficial to the readers.

***Reviewer #3:***

*There are no major issues with the execution of this study. No further experiments required.*

**Response:** We thank the reviewer for this comment!

**Minor Issues**

***Reviewer #1:*** *(No Response)*

***Reviewer #2:***

*1. Fig. 1C and 2B: The way this data is normalized to non-transduced cells is not fully clear to this reviewer. Does the data contain any information/correlation to the relative expression of RTP4 or the respective autofluorescent protein as a correlate?*

**Response:** We thank the reviewer for this comment. We normalized by dividing the frequency of NS5A^+^ cells in experimental samples by the frequency of NS5A^+^ cells in non-transduced, HCV-infected cells. We provide data in the supplementary data of this resubmission showing RTP4 expression (RT-qPCR + Western blotting of tagged constructs) and reporter fluorescence (flow cytometry) following transfection of 293T cells.

*2. Fig. 2, lines 140-160: I do not fully agree with the interpretation of data by the authors. Replacement of the murine DRV/TM domain by the hs counterpart dramatically impacts on the restricting function of mmRTP4 (construct 2 compared to M). Therefore, the conclusion that the ZFD is the only determinant of the difference is in my view not supported by the data. The only chimera widely resembling the restricting function of mmRTP4 is construct 6, harboring the N-terminus of the hhZFD, while constructs 7 and 8 have no restricting capacity. Overall, the data at this point support auxiliary roles of mmDRV/TM.*

**Response:** We thank the reviewer for this suggestion; we have added language to the text indicating an auxiliary impact of the murine DVR-TM region for HCV restriction.

*3. Fig. 4: Panels B and C are not referred to in the text. The data should be interpreted more explicitely, regarding the nature of induced genes. The text mentions non-infected controls, how do this data look in comparison? Are the genes induced also in absence of HCV infection? Is any of the induced gene products secreted and could account for the trans-inhibitory effect observed in Fig. 3D?*

**Response:** We thank the reviewer for pointing out this oversight. We cite and briefly discuss these panels in the relevant paragraphs of the revised manuscript (none of which, to our knowledge, are potential morphogens). We used samples transduced with a blank lentiviral vector as the baseline for examining mmRTP4- and hsRTP4-driven changes to gene expression, given that we wanted to avoid any nonspecific/confounding effects of lentiviral transduction. RNAseq data for the true-mock samples is available in the NCBI GEO submission attached to this manuscript (GSE292032; token “otmvkucyppqflmr”). For logistical reasons, we did not subject HCV-uninfected, RTP4-transduced samples to RNA sequencing.

*4. Fig. 5D: IF data on NS5A should be included to show that NS5A is expressed to similar levels in both conditions.*

**Response:** We thank the reviewer for this comment. We have included staining controls for NS5A, FLAG-hsRTP4, and FLAG-mmRTP4 within Figure 5 in the resubmission. We note that we deliberately selected an early timepoint for this experiment in order to capture the mmRTP4-NS5A interaction.

*5. Fig. 6D/E, 7D: How can HCV RNA be quantified up to several logs below LLOD? Formally, everything below LLOD should be non-detectable.*

**Response:** We thank the reviewer for this comment. We have modified the figure to place all data points at the LLOD for these figures.

*6. The authors claim throughout the manuscript, that mmRTP4 impacts on virion formation. However, mmRTP4 has no impact on extracellular titers and reduces intracellular titers (Fig. 3E/F). This phenotype could be fully explained by reduced RNA replication. Such mechanistic claims need to be supported by additional data (e.g. measuring HCV intracellular RNA, which should be unaffected, or using replicons) or avoided, e.g. by using more general terms (…impacts on viral replication)*

**Response:** We thank the reviewer for this suggestion. We agree to tone down our language related to the mechanism to instead assess that “murine RTP4 impacts viral replication”.

***Reviewer #3:***

*The legend to Figure 3 is not easy to decipher and could with benefit to the reader be reworked for better clarity. Moreover, the data point in green (panel C) is only legible at very high magni-fication due to similar values as other data points. Means to mitigate this obstruction could also improve the figure.*

**Response:** We agree with the reviewer on this point. Unfortunately, we have not been able to successfully address this in GraphPad in an aesthetic manner. We note similar issues visualizing red and green data points in panel D.
